# Disrupted Causal Connectivity Anchored on the Right Anterior Insula in Drug-Naive First-Episode Patients With Depressive Disorder

**DOI:** 10.3389/fpsyt.2022.858768

**Published:** 2022-05-18

**Authors:** Haiyan Xie, Qinger Guo, Jinfeng Duan, Xize Jia, Weihua Zhou, Haozhe Sun, Ping Fang, Hong Yang

**Affiliations:** ^1^Department of Psychiatry, The Fourth Affiliated Hospital, College of Medicine, Zhejiang University, Yiwu, China; ^2^Department of Radiology, The Fourth Affiliated Hospital, College of Medicine, Zhejiang University, Yiwu, China; ^3^Department of Psychiatry, The First Affiliated Hospital, College of Medicine, Zhejiang University, Hangzhou, China; ^4^School of Teacher Education, Zhejiang Normal University, Jinhua, China; ^5^Key Laboratory of Intelligent Education Technology and Application of Zhejiang Province, Zhejiang Normal University, Jinhua, China; ^6^School of Social Sciences, Heriot-Watt University, Edinburgh, United Kingdom; ^7^Department of Radiology, The First Affiliated Hospital, College of Medicine, Zhejiang University, Hangzhou, China

**Keywords:** major depressive disorder, right insula, causal connectivity, salience network, default mode network, central executive network, stroop test

## Abstract

**Object:**

Major depressive disorder (MDD) has been demonstrated to be associated with abnormalities in neural networks. However, few studies examined information flow in the salience network (SN). This study examined abnormalities in the causal connectivity between the SN and whole brain in drug-naive first-episode patients with MDD in the resting state.

**Methods:**

Based on the Diagnostic and Statistical Manual of Mental Disorders (DSM-5) diagnostic criteria, 23 drug-naive first-episode MDD patients and 20 matched healthy individuals were recruited and underwent a resting-state magnetic resonance scan. The acquired functional image data were preprocessed using resting-state functional magnetic resonance imaging (rs-fMRI) data analysis toolkit plus (RESTplus). Then, using the data processing & analysis for brain imaging (DPABI) software and a coefficient-based general component analysis method with the right anterior insula (rAI) as the region of interest (ROI), the causal connectivity of the SN with the whole brain and its correlation with cognitive and mental performance were examined in the resting state.

**Results:**

(1) The MDD group showed a significantly higher Hamilton Depression Rating Scale total score and significantly higher scores for anxiety, cognitive disturbance, and block factors compared with normal controls. (2) Compared with control: from whole brain to the rAI, the MDD group showed a lower causal connectivity in the left inferior frontal gyrus; from the rAI to the whole brain, the MDD group showed a lower causal connectivity in the right cingulate gyrus, the right precuneus, and extending to paracentral lobule but higher causal connectivity in the left inferior and middle frontal gyrus. (3) In the MDD group, from rAI to the whole brain, the causal connectivity values for the right cingulate gyrus/precuneus were negatively correlated with the score of Stroop Color-Word Test A, B, and C as well as interference times.

**Conclusion:**

Our results indicated disrupted causal connectivity among the default mode network (DMN), the central executive network (CEN), and SN in drug-naive first-episode MDD patients. Especially, our results suggest a unique role for rAI in the ordered or hierarchical information processing, presumed to include bottom-up and top-down reciprocal influences among the three networks in MDD.

## Introduction

Characterized as persistent negative emotions, a lack of motivation and changes in cognitive functions, major depressive disorder (MDD) is one of the most common mental disorders (1). According to the “Depression and Other Common Mental Disorders: Global Health Estimates” issued by the World Health Organization (WHO) in February 2017, the total number of people living with depression worldwide is 322 million. The World Health Organization ranked depressive disorders as the single largest contributor to non-fatal health loss (7.5% of all years lived with disability). Although depression does great harm to individuals, its pathogenesis largely remains unclear.

The brain is a complex network with extensive interconnections in structure and function among different parts of the brain. Functional coordination among brain regions requires the exchange of information among different regions. This information exchange plays an important part in complex cognitive processes (2). With the technique of resting-state functional magnetic resonance imaging (rs-fMRI), it was found that depression results from the dysfunction of multiple brain regions caused by connectivity abnormalities (3, 4). Previously, related studies have mainly focused on functional networks related to movement control, cognitive processing, emotional processing, etc., such as default mode network (DMN), central executive network (CEN), and salience network (SN) (3, 5–7).

The SN is mainly composed of the insular cortex, the dorsal side of the anterior cingulate gyrus, the amygdala, and the temporal lobe. It controls the signal transmission between the CEN and DMN, acting as a regulatory “switch,” such as attention to the external environment for the CEN and the monitoring of internal information for the DMN (8). According to the review by Menon, an important cause of the depressive disorder is likely to function abnormalities in salience information detection between the networks and the destruction of the internal and external information mapping functions, i.e., abnormal conversion between the DMN and CEN caused by anterior insular dysfunction in the SN, which in turn leads to abnormal behavioral responses to internal and external stimuli and events (6).

The SN may change with the emotion processing process, and the insula, as a core node of the SN, i.e., a cross-boundary region in processing cognitive functions, internal states, and emotional perceptions, plays a very important interactive role in the monitoring of endogenous stimuli and exogenous information (9). Therefore, in studies of the SN that employ the seed point-based functional connectivity method, the insular cortex is often used as the region of interest. The anterior insula (AI) is the control center that regulates the dynamic interactions involving brain networks and plays a very important role in autocorrelated mental activities involving external attention and internal orientation, having a right-side advantage (7). Several studies have linked the AI to both the processing of mutual feeling states and adaptive switching between functional networks, making this region an ideal candidate for exploring potential neural circuit dysfunction in emotional disorders (10, 11).

Causal connectivity refers to the direct or indirect causal influence of one brain area on another brain area (12, 13). Granger causality analysis (GCA) is a relatively data-driven analytical method for connectivity, analyzing the direction of information flow between brain regions based on the time series of information processing and thus enabling a description of resting-state brain networks (13, 14). A regression coefficient-based algorithm has been proposed in which signed regression coefficients are used to assess the Granger effect: a positive value indicates an excitatory effect and a negative value indicates an inhibitory effect or negative feedback (15). This method can more truly reflect brain activity, with low computational complexity, making the calculations easier. Using regression coefficient-based GCA, our previous research found that depression patients in the resting state exhibit abnormalities in the frontal lobe network, confirming that MDD patients have impaired top-down cognitive control (16).

Therefore, in this study, with coefficient-based GCA method and the right anterior insula (rAI) as the seed point, we examined the direction of information flow between the SN and the whole brain and their correlations with cognitive and mental performance in drug-naive first-episode MDD patients in the resting state. We hypothesized that there would be the abnormalities in the causal connectivity between the rAI and DMN or CEN meanwhile the abnormal GCA would be correlated with clinical psychological scale scores in MDD patients.

## Materials and Methods

### Subjects

#### Study Group

Twenty-three depression patients (seven men and 16 women; outpatients and inpatients) of the Psychiatry Department, First Affiliated Hospital, Zhejiang University School of Medicine, were included in the study (MDD group). According to the clinical interview and the Structured Clinical Interview for DSM, the diagnosis was unanimously ascertained by two licensed psychiatrists with intermediate professional titles. The inclusion criteria were as follows: met the diagnostic criteria for depression in “Diagnostic and Statistical Manual of Mental Disorders (DSM-5)” as well as the International Classification of Disease (ICD-10) diagnostic criteria for depression; first episode of depression and had not yet received treatment; education of junior high school or above; right-handed; and Hamilton Depression Rating Scale (HAM-D) (17-item) score ≥17 points.

#### Control Group

Twenty normal individuals (six men and 14 women), matched for age, education, and right-handedness, were recruited from hospital staff, students, and volunteers from nearby communities. The inclusion criteria were as follows: healthy adults without a history of mental illness or a family history of mental illness; education of junior high school or above; and HAM-D score ≤ seven points.

The general exclusion criteria were as follows: (1) treated depression; (2) a history of diseases with abnormal brain structure, epilepsy or significant medical illness; (3) any other mental disorders (except MDD in patients) after clinical interviews and Structured Clinical Interview screening; (4) any family history of mental illness; (5) a history of alcohol and drug abuse; (6) women pregnant, lactating or in a menstrual period, and (7) magnetic resonance imaging (MRI) contraindications, such as metallic implants, retractors or braces, and claustrophobia.

Moreover, patients with MDD usually have cognitive executive function impairment (17). Considering of immature or possibly declined cognitive function (18), 18–45-year-old patients were chosen as subjects. This study was approved by the local Medical Ethics Committee of First Affiliated Hospital of Zhejiang University, and each subject signed an informed consent form.

### Clinical Assessments and Neuropsychological Tests

The severity of mood symptoms was assessed using the 17-item HAM-D, which is categorized into six dimensions, i.e., anxiety, weight, cognitive disturbance, diurnal variation, block and sleep disorders. In this study, we only analyzed the scores for anxiety, cognitive disturbance, and block factors, as well as the HAM-D total score.

Two well-known neuropsychological tests, Stroop Color-Word Test (SCWT) (19) and Trail-making test (TMT) (20), were performed on each subject, including attention, memory, processing speed, behavior inhibition and executive function. The two tests have been proved to reveal cognitive deficits in patients with MDD in many studies.

The SCWT A and SCWT B were conducted to measure selective attention and processing speed, SCWT C and SCWT interference were used to measure behavioral inhibition and executive function. SCWT A asked subjects to read aloud three written colors printed in black ink as quickly as possible. SCWT B required the subjects to name the color as quickly as possible. Finally, the subjects were required to name the ink color of a color word as fast as possible to finish SCWT C. The color word was not the same as the ink color. The performance for each condition was recorded as the processing time per item in seconds. The reaction time difference in SCWT C relative to SCWT B is the SCWT interference. There were two subtasks: write and read aloud color names that differ from printed ink colors.

The TMT was divided into two parts, which were used to evaluate attention and executive function. For the TMT-A, subjects were asked to draw lines quickly to connect consecutive numbers. It measured visuo-spatial attention and performance speed. For the TMT-B, subjects were asked to connect numbers and letters alternately in ascending order as quickly as possible, the part measured mental flexibility, ability to shift attention, and strategy. Task completion was measured in seconds.

### Image Acquisition

The MRI data were collected on a Philips Achieva 3.0 T scanner in the First Affiliated Hospital of Medical College of Zhejiang University. The participants were asked to lie on the scanner with their eyes closed, do not think about anything in particular, and do not fall asleep. An echo-planar imaging (EPI) sequence was used for functional magnetic resonance imaging. The parameters were as follows: repetition time (TR), 2000 ms; echo time (TE), 35 ms; flip angle, 90°; slice thickness/interval, 5.0/1.0 mm; and number of volumes (or time points), 200. A total of 24 transverse slices were used to cover the whole brain, with all slices arranged in parallel with the anterior-posterior commissure. A gradient-echo sequence was also used to obtain high-resolution T1-weighted structural MRI images. The parameters were as follows: TR/TE, 8/4 ms; flip angle, 8°; matrix size, 240 × 240; slice thickness, 1 mm; and field of view, 240 mm × 240 mm.

### Imaging Data Preprocessing

Preprocessing of rs-fMRI data was performed using Resting-State fMRI Data Analysis Toolkit (RESTplus) (http://restfmri.net/forum/RESTplus) (21), including (1) removal of the first 10 time points for steady magnetization and participant adaptation; (2) slice timing correction; (3) head motion correction; (4) performing spatial normalization to the Montreal Neurological Institute (MNI) space by using new segment to the structural images (resampling voxel size = 3 mm × 3 mm × 3 mm); (5) spatial smoothing using an isotropic Gaussian kernel of 6 mm full width at half maximum (FWHM); (6) removal of the linear drift within the time series; (7) regressing out the effects of head movement (Friston 24 parameter model), the cerebrospinal fluid (CSF) signal noise, and the white matter signal noise from the fMRI data (22); and (8) band-pass filtering (0.01–0.08 Hz). One participant in the healthy control group was excluded because head motion is larger than 3.0 mm or 3.0°.

As a powerful approach, GCA may be confounded by varied hemodynamic response functions (HRFs) across the brain. We then employed a blind deconvolution method to control the varied HRF which has been demonstrated in detail by Wu et al. (23). The method considers the HRF modeled from the spikes of large amplitude Blood Oxygen on Lever Depending (BOLD) signal based on the assumption that such spikes represent spontaneous neural activities. The lack of explicit inputs for modeling signal dynamics and estimating the underlying neural signal remains a challenge for rs-fMRI data compared with task-based fMRI (22, 23). It has been argued that this makes the representation of BOLD-level causal influences to underlying neural-level causal influences more difficult (24). The approach solves these problems by performing deconvolution of the HRF with the BOLD signal, which allows the extraction of neuronal variables from the signal, thus being closer to neural-level causal models. In accordance with Wu et al. (23), a threshold of 1.2 standard deviations was used for determining spikes and 15 s for a maximum lag between a neural event and BOLD response.

### Granger Causality Analysis

To study the influence of depression on the brain, we used a sphere centered at the rAI [MNI coordinate (51, 33, −3)] with a 6-mm radius as the seed region for the GCA (25). The driving effects and feedback effects were calculated between the time series of seed region and the time series of each voxel in the whole brain by using GCA in this study. The bivariate coefficient GCA was performed using REST softwarche (http://www.restfmri.net) (26). Granger causality estimates the causal effect of the seed-to-whole-brain and whole-brain-to-seed. In the current study, the times series of the seed was defined as X, and the time series of each voxel was Y. A positive coefficient from X to Y indicates that X has a Granger causal influence on Y (i.e., positive influence). Similarly, a negative coefficient from X to Y suggests that the activity in region X exerts an opposing directional influence on the activity in region Y (i.e., negative influence). The coefficient maps for all subjects were *z*-transformed to improve normality.

### Statistical Analysis

Two-sample *t*-tests were performed to compare the GCA maps between patients with depression and the healthy control group. All the statistical analyses were conducted within a gray matter mask (using a gray matter probability template with a threshold of 0.2). The Gaussian random field theory correction was used for the *T*-maps with a threshold of voxel *p* < 0.05, cluster *p* < 0.05, and edge connected. The analyses were performed using data processing & analysis for brain imaging (DPABI) (27). Finally, we extracted the mean GCA value in the areas of significant difference in gray matter, Pearson correlation analysis was performed between the mean GCA value and the score of HAM-D, SCWT, and TMT in patients.

## Results

### Demographic, Clinical, and Neuropsychological Test Results

As shown in Table 1, there is no significant difference between the two groups in age, sex, and education level (*p* > 0.05). In addition, there was no significant difference in the SCWT and TMT between the two groups (*p* > 0.05), but a significant difference in the total HAM-D score (*t* = 18.41, *p* < 0.01) and in the scores for anxiety (*t* = 11.69, *p* < 0.01), cognitive disturbance (*t* = 7.50, *p* < 0.01) and block factors (*t* = 19.31, *p* < 0.01) between the two groups.

**Table 1:** Demographics and clinical characteristics of the subjects.

**Variables (mean ±SD)**	**MDD (*n* = 23)**	**NC (*n* = 20)**	***P*-value**
Age (years)	31.526 ± .86	30.107 ± .52	0.449^b^
Gender (male/female)	7/16	6/14	0.975^a^
Education (years)	12.133 ± .86	12.403 ± .13	0.741^b^
Illness duration (months)	11.871 ± 0.92	/	/
HAM-D score	25.095 ± .57	1.251 ± .68	0.000^b^
Anxiety factors	7.392 ± .37	0.701 ± .03	0.000^b^
Cognitive disturbance factors	4.482 ± .59	0.100 ± .30	0.000^b^
Block factors	8.391 ± .75	0.350 ± .67	0.000^b^
TMT A	58.872 ± 9.05	59.204 ± 3.06	0.976^b^
TMT B	110.395 ± 6.89	106.858 ± 5.97	0.873^b^
SCWT A	49.152 ± 7.68	45.961 ± 2.87	0.733^b^
SCWT B	74.522 ± 3.47	64.052 ± 4.20	0.131^b^
SCWT C	115.03 ± 3.44	104.42 ± 5.55	0.295^b^
SCWT interference	40.391 ± 7.67	40.301 ± 7.73	0.761^b^

*MDD, major depressive disorder; NC, normal control; HAM-D, Hamilton Depression Rating Scale; TMT, Trail-making test; SCWT, Stroop Color-Word Test*.

a *The P-value was obtained by chi-square test*.

b *The P-values were obtained by Mann–Whitney U test*.

### Granger Causality Analysis

From the whole brain to the region of interest (ROI) (Y to X), in the left inferior frontal gyrus (lIFG), causal connectivity in the MDD group was lower than that in the control group. The MDD group did not show any region with causal connectivity that was higher than that in the control group.

**Figure 1:**
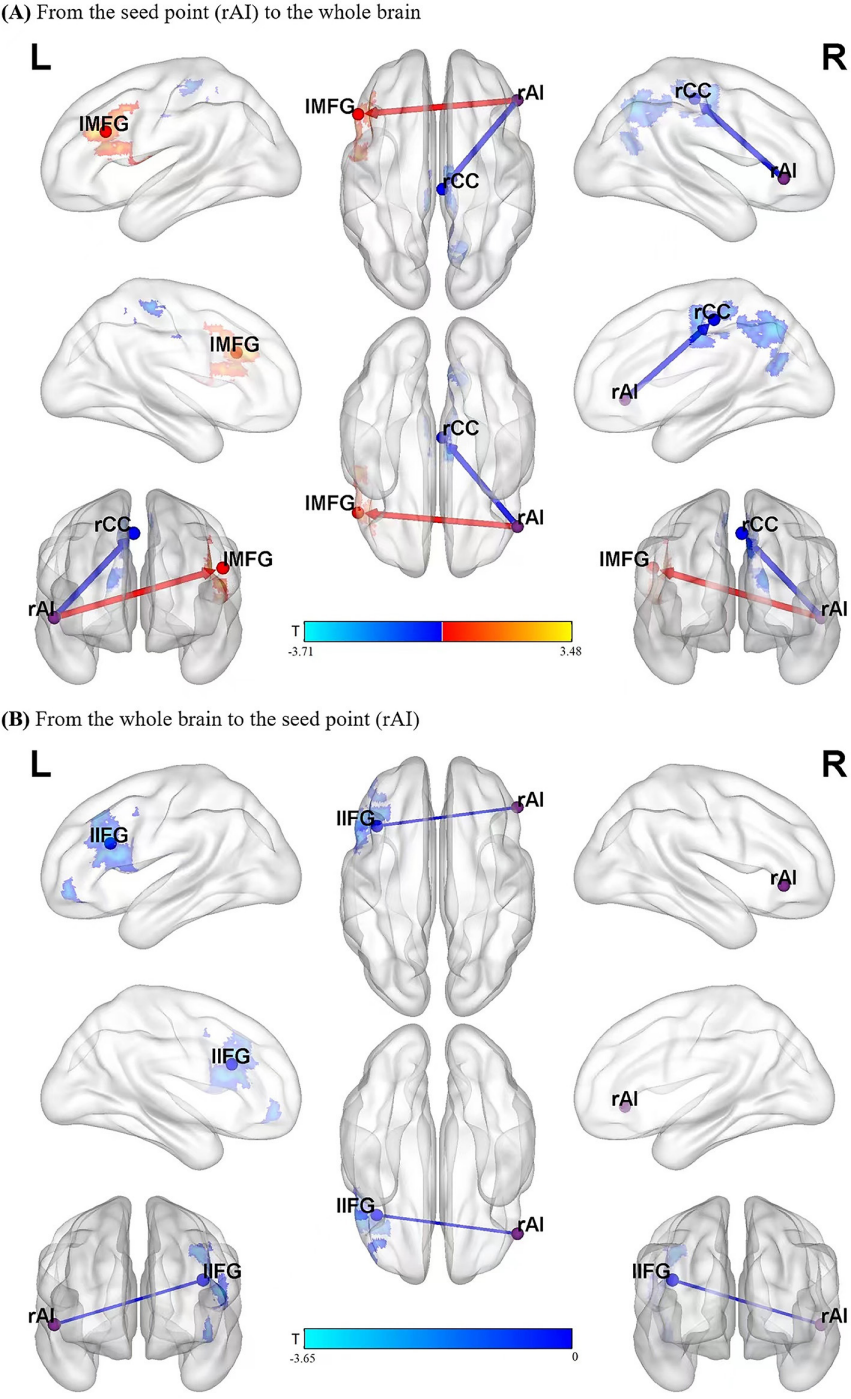
Blue areas show brain regions where MDD patients had reduced causal effects than controls, while red-yellow colored areas show brain regions where patients had increased causal effects than controls. The color bar represents *t*-values. The significant results of GCA between the two groups **(A,B)**. rAI, right anterior insula; rCC, right cingulate cortex; lMFG, left middle frontal gyrus; lIFG, left inferior frontal gyrus.

From the ROI to the whole brain (X to Y), for the following regions, the causal connectivity in the MDD group was lower than that in the control group: right cingulate cortex (rCC), right precuneus (rPreC), and paracentral lobule (PCL); from the ROI to the whole brain (X to Y), in the left middle frontal gyrus (lMFG) and lIFG, the causal connectivity in the MDD group were higher than that in the control group. It was shown in Table 2 and Figure 1.

**Table 2:** Significant group differences in Granger causality analysis.

**Regions**	**MNI (x, y, z)**	**Cluster size (mm^**3**^)**	***t*-value**	***P*-value**
**Causal inflow to the rAI from the whole brain**				
lIFG	−51, 24, 27	114	−3.7167	<0.05
**Causal outflow from the rAI to the whole brain**				
rCC	3, −24, 48	56	−3.8407	<0.05
rPreC	3, −24, 48	136	−3.8407	<0.05
PCL	3, −24, 48	77	−3.8407	<0.05
lIFG	−39, 21, 24	64	3.4310	<0.05
lMFG	−39, 21, 24	104	3.4310	<0.05

*rAI, right anterior insula; rCC, right cingulate cortex; rPreC, right precuneus; PCL, Paracentral lobule; lIFG, left inferior frontal gyrus; lMFG, left middle frontal gyrus*.

### Correlation Analysis

**Figure 2:**
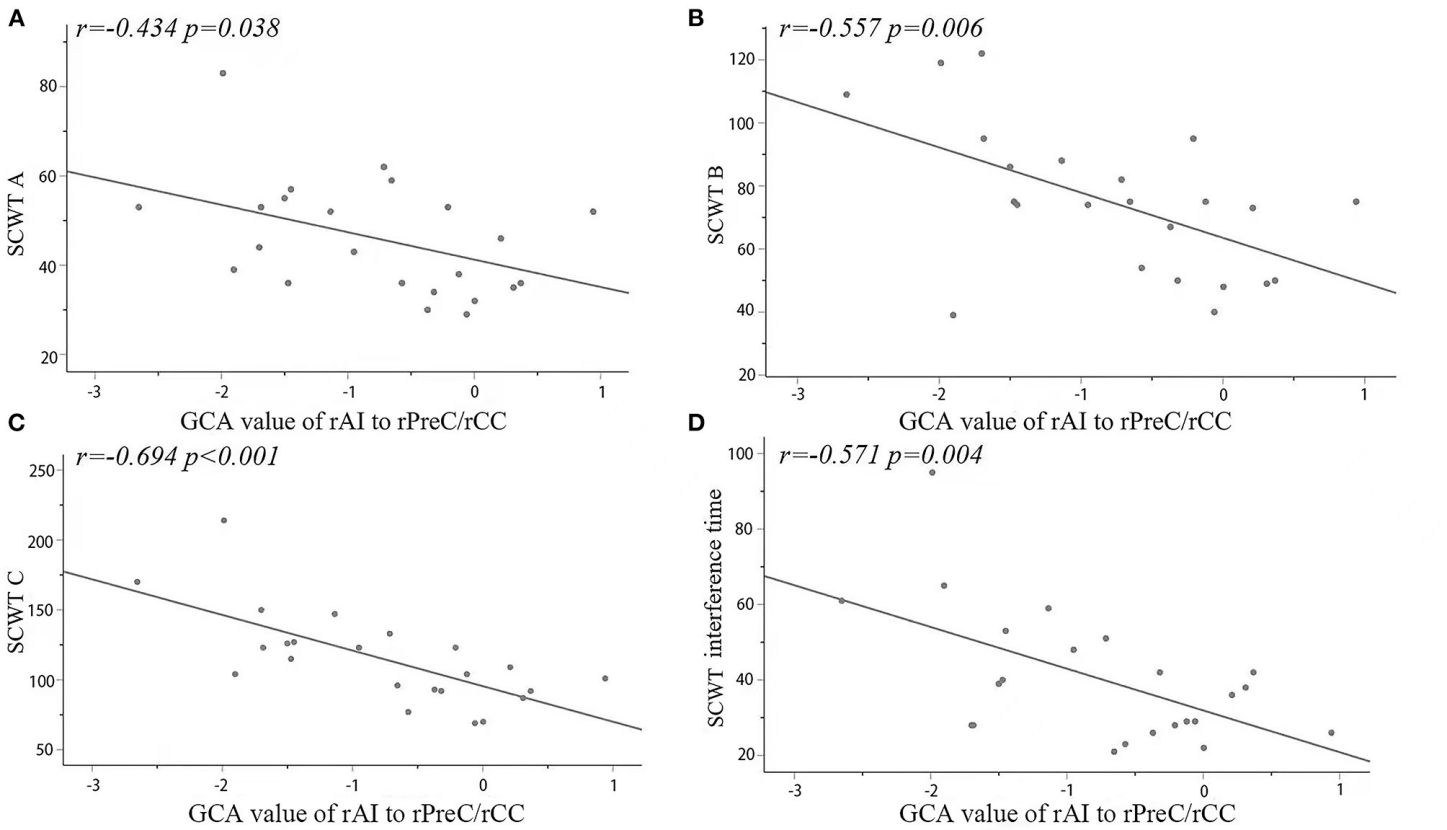
Significantly negative correlations between the causal connectivity values and the SCWT **(A–C)** and interference times in the rPreC/rCC from the seed point to the whole brain in the MDD group. The correlations of the GCA values with the SCWT in the MDD group **(D)**. rAI, right anterior insula; rPreC, right precuneus; rCC, right cingulate cortex; SCWT, Stroop Color-Word Test.

In the MDD group, the signals within the cluster range that were statistically significant between two groups were directly extracted and analyzed based on the correlation with the scores from the scales, the results showed that from the seed point to the whole brain, the causal connectivity values for the right cingulate gyrus/right precuneus were negatively correlated with SCWT A, SCWT B, SCWT C, and SCWT interference time but not significantly correlated with the HAM-D total score and the score for each factor. They were shown in Figure 2.

## Discussion

In this study, with a seed point-based GCA, we found that the causal connectivity from lIFG (a key node of the CEN) to the rAI (a key node of the SN) in the MDD was lower than that in the controls. Moreover, the causal connectivity from the rAI to rCC, rPreC, and PCL (the key nodes of the DMN) in the MDD group was lower than that in the normal group, while the causal connectivity from the rAI to lMFG and lIFG in the MDD was higher than that in the normal group. The results suggest aberrant information flow between the SN and the DMN or CEN in drug-naive first-episode MDD patients. Moreover, the decrease in the causal connectivity from the rAI to the components of the DMN (rPreC/rCC) was negatively correlated with the SCWT A, B, and C and interference time.

In recent years, the DMN, CEN, and SN have been hotspot networks of depressive disorder. The ability to switch neural resources between DMN, SN, and CEN has been considered to be the key mechanism of adaptive emotion regulation (28). DMN is associated with spontaneous brain activities involving episode-related memory retrieval and the monitoring of the external environment and exogenous stimuli as well as the state of the self-reflection and the regulation of endogenous information, which in turn is related to the advanced cognitive perception and emotional processing (29). The CEN is closely related to attention and working memory, and controls the network's involvement in cognitive tasks, playing an important role in adaptive cognitive control. The SN is mainly involved in assessing external affairs and responding accordingly to connect to the CEN and DMN. The study (30) found that MDD patients show connectivity abnormalities in internal functions of the SN and that the decrease in functional connectivity within the rAI of the SN is correlated to the severity of depressive symptoms and abnormal DMN/CEN interactions. Another study (31) reported that functional connectivity increased between the SN and CEN as well as the dynamic interaction between the DMN and CEN decreased for MDD. However, these studies on functional connectivity did not take into account the direction of information flow between the triple network model, which is important to understand the causality of DMN, CEN, and SN in MDD.

In this study, we found that abnormal the causal connectivity between the rAI and the frontal lobe, including decreased from the lIFG to the rAI, but increased from the rAI to lMFG and lIFG. The frontal lobe lesions can change cognitive function and behavioral and decision-making capacity, while affecting emotion and mood. Abnormalities in the functional connectivity of the frontal cortex in patients with depression have been reported in many imaging studies (16, 32–35). Our result showed that the unidirectional causal effect of the lIFG on the rAI was reduce, it suggested the patients decreased externally oriented task information flow (top-to-down), and impaired the lIFG function that related to higher demand or a specific cognitive process (36). The unidirectional causal effect of the rAI on the lMFG and lIFG was increased in patients in our study, which may suggest, in the early stage of the MDD, as feedback or compensatory, increased self-referential cognition information flow (down-to-top) to general reorganization of brain functioning. The frontal lobe is a key part of the CEN. According to the triple model proposed by Menon (6), SN might play a central role in mediating the transformation of the functional connectivity in CEN, which is related to the externally oriented task, especially the region rAI in SN. Our results indicated the impaired triple model in MDD.

We found that the causal connectivity from rAI to DMN nodes (rCC/rPreC and PCL) is decreased. The precuneus/cingulate cortex, which belongs to the posterior sub-network, is an important part of DMN. It has been reported that a change in the resting-state functional connectivity between the rCC and rAI indirectly reflects changes in the emotional regulation function of MDD patients (37). Precuneus as a key node of DMN has been reported to be participated in the self-consciousness and self-related mental representations, the dysfunctions of precuneus were related to depressive symptoms, such as somatic complaints, negative bias in interpreting bodily feedback and negative emotional memory, some key features of MDD (38). Yuen GS et al. adopted the rAI as the seed point and found that the rAI and bilateral precuneus had abnormal functional connectivity in the depression group (39). In addition, we also found that the causal connectivity from the rAI to the PCL was lower in the MDD group than that in the controls. The PCL (an important part of the CEN)participates in a variety of complex functions and is critical for somatic motor processing, regulating subjective well-being, and emotion processing (40). The decline of information flow from the rAI to the PCL may be one of the pathological mechanisms that cause non-specific physical symptoms in patients with depression. In our study, decreased causal connectivity from rAI to rCC/rPreC and PCL suggested the decreased down-top information flow, which may be related to the breakdown of this presumed hierarchical processing system from sensory to higher cognitive regions, mediated by core limbic regions (e.g., insula) in MDD. In patients, those results also proved the impaired triple model (6) – that SN has a core role in mediating the conversion of the functional connectivity between the DMN, which is related to self-referential cognition.

We found that the two groups did not differ significantly on the SCWT and TMT, i.e., in terms of the psychometric measurements of cognitive functions using the SCWT and TMT, the scores for the two groups did not differ significantly. This was just similar to our GCA results – decreased causal connectivity from the frontal lobe to the rAI, but increased causal connectivity from the rAI to the frontal lobe which suggested, in the early stage of the MDD, like feedback or compensatory, increased self-referential cognition function to general reorganization of brain functioning. In addition, the higher education level in our subjects might be another influencing factor. The study has shown that individuals with a high level of education have greater cognitive reserves than those with a low level of education, enabling those individuals to better withstand brain damage and maintain function (41).

A meta-analysis conducted by McDermott et al. (42) confirmed that depression severity is positively correlated with cognitive deficits in terms of episodic memory and processing speed. We observed that the change in the GCA from the rAI to the rPreC/rCC was negatively correlated with the Stroop test cards A, B, and C and interference times. Subtest A measures automatic processing and the flexibility of attention; subtest B measures controlled processing and the concentration of attention; and subset C measures the selective attention and response inhibition of executive functions. Our results suggested that, in the early stage of the MDD, the impairment in cognitive function might be the beginning with attention concentration, selective attention of executive function, and response inhibition function, and they were progressed with the severity of the depression.

In summary, we found the abnormal information flow among DMN, CEN, and SN in drug-naive MDD patients. It suggested that the rAI had an important role in orderly or hierarchical information processing, which was considered to include bottom-up (sensory to multimodal) and top-down (multimodal to sensory) reciprocal influences among the three networks. Our results also suggest that in the early stage of MDD, as feedback or compensatory, MDD patients can increase self-referential cognition function to reorganize brain function.

## Limitations

This study used depression patients who had only one episode of depression, had not taken any medication, and did not have other mental illnesses, thus avoiding interference from factors, such as past medication use and chronic disease. However, there are still some limitations. First, the sample size was small, which may cause biases in the results. Second, the seed point-based choice has a substantial impact on the results, and the selection criteria are highly arbitrary and lack unified standards; therefore, the bias related to the brain area of interest generates incomplete results and results that are dependent on assumptions. Lastly, similar to most rs-fMRI studies, we used a TR of two s, leading to very limited time resolution. This problem can be solved by developing sub-second whole brain scan sequences.

## Conclusion

In conclusion, our results indicated disrupted causal connectivity among DMN, CEN, and SN in the first-episode patients with depressive disorder. Especially, our results showed that the anterior insula had a unique role in orderly or hierarchical information processing, which was considered to include bottom-up (sensory to multimodal) and top-down (multimodal to sensory) reciprocal influences among the three networks in depressive disorder. Our results also suggested that the information flow involves in the execution, memory, and attention function of the brain from SN to DMN was decreased with the severity of the depression.

## Data Availability Statement

The raw data supporting the conclusions of this article will be made available by the authors, without undue reservation.

## Ethics Statement

The studies involving human participants were reviewed and approved by Ethical Committee approval was received from the Ethics Committee of First Affiliated Hospital, Zhejiang University School of Medicine (Approval No. 2016-27). The patients/participants provided their written informed consent to participate in this study.

## Author Contributions

HX designed the research, collected samples, and wrote the original of manuscript. QG, JD, WZ, and PF collected samples, supervised data, and conducted quality control. XJ and HS analyzed the data. HY supervised and designed the research, collected samples, and revised the manuscript. All authors contributed to the article and approved the submitted version.

## Funding

This study was supported by the Basic Public Welfare Research Program of Zhejiang Province (Grant No. LGF20H090013), the Key Research and Development Program, Ministry of Science and Technology of People's Republic of China (Grant No. 2019YFC0121003), the Science Technology Department of Zhejiang Province (Grant No. 2017C33096), and the Health and Family Planning Commission of Zhejiang Province (Grant No. 2015107509).

## Conflict of Interest

The authors declare that the research was conducted in the absence of any commercial or financial relationships that could be construed as a potential conflict of interest.

## Publisher's Note

All claims expressed in this article are solely those of the authors and do not necessarily represent those of their affiliated organizations, or those of the publisher, the editors and the reviewers. Any product that may be evaluated in this article, or claim that may be made by its manufacturer, is not guaranteed or endorsed by the publisher.
